# Association Between Oral Microbiota Dysbiosis and the Risk of Dementia: A Systematic Review

**DOI:** 10.3390/dj13060227

**Published:** 2025-05-22

**Authors:** Alain Manuel Chaple-Gil, Meylin Santiesteban-Velázquez, Joaquín Juan Urbizo Vélez

**Affiliations:** 1 Facultad de Ciencias de la Salud, Universidad Autónoma de Chile, Santiago 7500912, Chile; 2 Instituto de Ciencias Básicas y Preclínicas (ICBP) “Victoria de Girón”, Universidad de Ciencias Médicas de La Habana, La Habana 11300, Cuba; santiestebanm00@gmail.com; 3 Facultad de Estomatología de La Habana “Raúl González Sánchez”, Universidad de Ciencias Médicas de La Habana, La Habana 10400, Cuba; joaquin.urbizo@infomed.sld.cu

**Keywords:** Alzheimer’s disease, cognitive dysfunction, mild cognitive impairment, oral microbiome, microbiota, oral health

## Abstract

**Background/Objectives**: Growing evidence suggests that oral microbiota dysbiosis may contribute to the development of systemic conditions, including neurodegenerative diseases. This dysregulation promotes immunoinflammatory responses that are increasingly associated with dementia. This systematic review aimed to evaluate the association between oral microbiota dysbiosis and the risk of dementia in older adults. **Methods**: Eligible studies evaluated oral microbial composition using validated methods such as genetic sequencing, bacterial culture, or metagenomic analysis. Following PRISMA guidelines and a PICO framework, the review included cohort, case–control, and cross-sectional studies. Searches were conducted across PubMed, Scopus, Web of Science, Embase, and Cochrane Library. Two independent reviewers screened and selected studies, resolving disagreements through a third evaluator. **Results**: This systematic review revealed that *Tannerella forsythia*, *Fusobacterium nucleatum*, *Porphyromonas*, *Prevotella*, *Leptotrichia*, *Fusobacteriota*, *Peptostreptococcaceae*, and *Candida* spp. were consistently associated with Alzheimer’s disease and mild cognitive impairment, indicating their potential role in neurodegeneration. In contrast, *Streptococcus gordonii*, *Gemella haemolysans*, *Rothia*, *Neisseria*, and *Haemophilus* were reduced in cognitively impaired individuals, suggesting a link with healthy cognition. Studies also showed decreased microbial diversity in Alzheimer’s disease and the possible modifying effect of the APOE4 allele. Oral health interventions improved microbial composition and slowed cognitive decline, supporting the diagnostic and therapeutic potential of oral microbiota modulation. **Conclusions**: The findings suggest that oral microbiota dysbiosis may not only result from cognitive decline but also contribute to its pathogenesis. Future studies with larger and more diverse cohorts are recommended to validate these associations.

## 1. Introduction

The oral microbiota is defined as the community of microorganisms comprising bacteria, fungi, viruses, archaea, and protozoa that inhabit the oral cavity and form a complex ecosystem in constant interaction with the host. Under normal conditions in a healthy individual, a balanced relationship is maintained, referred to as microbial homeostasis or eubiosis, characterized by the predominance of beneficial microorganisms over pathogenic ones [1,2].

This eubiotic balance can be disrupted by various factors, shifting from mutualism or commensalism to a parasitic or pathogenic state in which microorganisms promote disease in the host. This imbalance, known as dysbiosis, can be associated with genetic predispositions, lifestyle habits, dietary patterns, and the use of certain medications [3,4].

When dysbiosis occurs, pathogenic bacteria may outcompete beneficial species, potentially leading to disease. This dysfunction can affect not only the local oral tissues but also distant organs. Importantly, oral dysbiosis is considered part of whole-body dysbiosis, which includes microbial imbalances in the gastrointestinal tract, skin, lungs, and genitourinary system, each with distinct characteristics and roles in health and disease [5].

Emerging evidence has demonstrated that oral dysbiosis can have far-reaching systemic consequences beyond the oral cavity. Bacterial pathogens and inflammatory mediators originating from periodontal infections may disseminate hematogenously, leading to colonization or inflammation of distant organs such as the heart, lungs, pancreas, and brain [6,7]. Furthermore, chronic periodontitis and other forms of oral dysbiosis contribute to a persistent low-grade systemic inflammatory state, characterized by elevated circulating cytokines and acute-phase reactants, which have been implicated in the pathogenesis of a wide range of systemic conditions including cardiovascular diseases, diabetes mellitus, rheumatoid arthritis, and neurodegenerative disorders such as Alzheimer’s disease (AD) [8,9]. This dual mechanism of microbial translocation and chronic systemic inflammation underscores the importance of investigating the potential causal links between oral health and systemic diseases. Strengthening this understanding may offer novel preventive and therapeutic strategies that target oral health as a modifiable risk factor for systemic conditions.

In addition, oral dysbiosis can induce mild yet persistent endotoxemia, as pathogenic microorganisms and their by-products can enter the bloodstream, triggering chronic systemic inflammation and immune dysregulation. This sustained inflammation has been linked to an increased risk of cardiovascular and neurodegenerative diseases, including dementia, with growing evidence supporting its role in the pathogenesis of these conditions [10,11,12,13].

Oral microbiota dysbiosis has drawn increasing scientific attention due to its potential link with various systemic diseases, including different forms of dementia in older adults. Recent studies suggest that certain oral bacteria may influence brain health. For instance, one study found that individuals with mild cognitive impairment (MCI) and a predominance of *Prevotella* in their oral microbiome were at higher risk of developing dementia. Conversely, a greater presence of *Neisseria* was associated with better memory and executive function [14]. In other hand, *Porphyromonas gingivalis*, a key pathogen in chronic periodontitis, has been identified in the brains of patients with AD. This bacterium produces toxic enzymes capable of damaging neurons, supporting the hypothesis of a connection between oral health and neurodegeneration [15].

Popesco et al. [16] further reinforce this view, highlighting that *P. gingivalis* is markedly altered in Alzheimer’s disease and contributes to microglial activation and cytokine production. Similarly, Sritana and Phungpinij [17], through their analysis of salivary markers in older adults, suggest that oral microbiota dysbiosis may be a valuable indicator for identifying individuals at risk of Alzheimer’s disease and MCI. However, they also note that the relationship between oral microbiota composition and neurodegenerative diseases remains poorly understood.

### 1.1. The Role of the Apolipoprotein

The Apolipoprotein E (APOE) gene is a well-established genetic factor influencing the risk of late-onset AD. Among its three common allelic variants (ε2, ε3, and ε4), the APOE ε4 allele has been consistently associated with an increased risk and earlier onset of AD. Individuals carrying one copy of APOE ε4 have an approximately threefold increased risk, while homozygous carriers face up to a 12-fold higher risk compared to non-carriers. APOE4 influences lipid metabolism and promotes the accumulation and impaired clearance of β-amyloid plaques, a neuropathological hallmark of AD. Emerging evidence also suggests that APOE4 may alter neuroinflammation and the integrity of the blood–brain barrier, further exacerbating neurodegeneration. Given the established association between oral microbiota dysbiosis and systemic inflammatory pathways, the presence of APOE4 may act as a biological modifier that enhances susceptibility to dementia through both amyloid-dependent and inflammatory mechanisms. Therefore, understanding the interaction between oral dysbiosis and APOE4 status is critical to elucidating potential pathways linking oral health and cognitive decline [18,19].

These findings underscore the importance of maintaining good oral health not only to prevent local conditions such as periodontitis but also to reduce the risk of systemic diseases, including dementia in older adults. Continued investigation into the mechanisms and implications of oral dysbiosis may open new avenues for the prevention, diagnosis, and treatment of neurodegenerative diseases with profound impacts on the aging population.

### 1.2. Research Question

What is the association between oral microbiota dysbiosis, assessed through genetic sequencing, bacterial culture, or metagenomic analysis, and the risk of dementia in older adults (≥50 years), compared to individuals with normotypic oral microbiota?

### 1.3. Objective

To evaluate the association between oral microbiota dysbiosis and the risk of dementia in older adults (≥50 years) by systematically reviewing observational studies that compare individuals with and without dementia, analyzing the microbial composition through validated sequencing, bacterial culture, or metagenomic methods.

## 2. Materials and Methods

### 2.1. Study Design

A systematic review was conducted following the guidelines of the PRISMA [20] (Preferred Reporting Items for Systematic Reviews and Meta-Analyses) methodology to ensure transparency and comprehensiveness. The study protocol was registered in PROSPERO with the ID CRD420251002818 platform, ensuring the standardization and replicability of the methodology employed.

### 2.2. Eligibility Criteria

#### 2.2.1. Inclusion Criteria

Observational studies investigating the association between the oral microbiota and the risk of dementia in older adults were included. Studies meeting the following criteria were included:

- Population: Older adults (≥50 years) with a dementia diagnosis.
- Intervention: Evaluation of the oral microbiota using genetic sequencing methods, bacterial culture, metagenomics, quantitative PCR, or other molecular analyses.
- Comparator: Individuals without a dementia diagnosis.
- Outcome: Measurement of the association between oral dysbiosis and the risk of dementia, cognitive decline, or neuroinflammatory biomarkers.
- Study Design: Observational studies (cohort, case–control, cross-sectional) analyzing the relationship between oral microbiota and dementia.

#### 2.2.2. Exclusion Criteria

Studies that did not assess the oral microbiota using validated methods were excluded to ensure the inclusion of research adhering to appropriate analytical standards. Additionally, studies conducted on animal models or in vitro were not considered, as this review focused exclusively on human populations. Narrative reviews, editorials, letters to the editor, and single-case studies were also excluded, as these types of publications do not provide relevant primary data for quantitative analysis. Lastly, studies that did not include a comparison between individuals with and without dementia were discarded, as the presence of an adequate control group was essential to evaluate the association between the oral microbiota and dementia.

The studies were grouped for synthesis according to cognitive diagnosis and by microbiota evaluation method.

### 2.3. Search Strategy

A comprehensive search was conducted in the PubMed, Scopus, Web of Science, Embase, and Cochrane Library databases to identify relevant studies. MeSH terms and keyword combinations related to the oral microbiota, dementia, and microbiological analysis methods were used. The search included terms such as “oral microbiome”, “oral dysbiosis”, “periodontal bacteria”, “dementia”, “Alzheimer’s disease”, and “cognitive decline”, among others. The last search was conducted on 19 February 2025. Filters were applied to exclude studies conducted on animal models and narrative reviews. Specific search formulations used in each database, as well as the applied filters, are detailed in Table 1.

### 2.4. Study Selection

A structured process was followed for study selection. Initially, results obtained from the databases were integrated into Rayyan^®^ and EndNote^®^, where duplicates were removed. Subsequently, two independent reviewers conducted a title and abstract screening to determine the relevance of each study. Studies that met the inclusion criteria proceeded to a full-text review phase, where their eligibility was verified based on the established criteria. In cases of disagreement between reviewers, a third evaluator resolved discrepancies.

The screening process for this systematic review will be conducted in two phases to ensure rigor and minimize bias. In Phase 1, two independent reviewers (A.M.C.-G. and M.S.-V.) assessed the titles and abstracts of all retrieved studies to determine their relevance based on the predefined inclusion criteria. Studies that did not meet these criteria were excluded at this stage.

In Phase 2, the full texts of all potentially eligible studies were retrieved for a more detailed evaluation. Each study was independently reviewed by both reviewers (A.M.C.-G. and M.S.-V.) to confirm its eligibility. In cases where there was a disagreement regarding the inclusion of a study, the issue was resolved through discussion. If consensus was not reached, a third reviewer (J.J.U.V.) was consulted to make the final decision.

This two-step process ensured that only high-quality, relevant studies were included in the review, enhancing the reliability and validity of the findings. To ensure consistency in study selection, calibration between reviewers was conducted before the screening process using Cohen’s kappa coefficient to measure inter-rater agreement. A moderate to high agreement was considered acceptable if the kappa value exceeded 0.60, with adjustments to criteria and methodology made for lower values. After calibration, the obtained Cohen’s kappa coefficient was 0.85, indicating excellent agreement among reviewers and ensuring high reliability in the selection of studies included in the review.

### 2.5. Data Extraction

Data were extracted independently by two reviewers (M.S.-V. and J.J.U.V.). No automation tools or contact with authors for missing data were used. The data extraction process was carried out using a standardized form designed to collect key information from each selected study. General data such as author name, year of publication, country, study design, and sample size were recorded. Additionally, detailed information on the characteristics of the included population was collected, specifying the number of patients with and without cognitive impairment, the type of dementia evaluated, and the neuropsychological scales used for diagnosis.

Regarding the oral microbiota, the employed analytical methods were documented, including polymerase chain reaction (PCR) and 16S rRNA or rDNA sequencing (Illumina MiSeq). The type of analyzed sample was also recorded, distinguishing between saliva (stimulated or unstimulated), subgingival plaque, and gingival crevicular fluid. The identified bacterial species associated with dysbiosis were classified into those that showed an increase and those that showed a reduction in individuals with cognitive impairment.

Finally, the main reported outcomes were extracted, including the association between specific bacterial taxa and cognitive decline. The studies reported alterations in microbial diversity, with increases in bacteria such as *Tannerella forsythia*, *Fusobacterium nucleatum*, *Veillonella*, *Porphyromonas*, *and Leptotrichia wadei*, while reductions were observed in species such as *Porphyromonas gingivalis*, *Capnocytophaga*, *Gemella haemolysans*, *and Streptococcus gordonii*. These findings suggest a potential link between oral microbiota dysbiosis and cognitive decline.

### 2.6. Risk of Bias Assessment

The Risk of Bias 2 (ROB 2) tool was used to assess the risk of bias in the included studies, and it was assessed independently by two reviewers (M.S.-V. and J.J.U.V.). ROB 2 evaluates five key domains: (1) bias due to assignment processes, (2) bias due to deviations from intended intervention, (3) bias due to missing outcome data, (4) bias in the measurement of outcomes, and (5) bias in the selection of reported results. Each domain was classified as “low risk”, “some concern”, or “high risk”, allowing for a structured assessment of the methodological quality of the studies. In cases of disagreement between reviewers, a third evaluator (A.M.C.-G.) resolved discrepancies.

Additionally, sensitivity analyses were conducted, excluding studies with a high risk of bias to evaluate the robustness of the results. To detect potential publication bias, funnel plots and Egger’s test were used to identify asymmetries that might indicate the presence of such bias.

### 2.7. Data Processing

For data analysis, RStudio^®^ version 2024.12.1 Build 563 was used. Structured methods were applied for the systematization and screening of the information extracted from the articles, ensuring the coherence and quality of the collected data.

The information extraction followed a standardized protocol, identifying and organizing relevant data from each study according to predefined criteria. Subsequently, a quality and relevance assessment of the studies was conducted using specific tools, allowing for an objective and reproducible classification.

### 2.8. Ethical Considerations

As this was a systematic review based on published studies, ethical approval was not required.

## 3. Results

A comprehensive literature search was conducted across five electronic databases, PubMed (*n* = 46), Scopus (*n* = 3717), Web of Science (WOS) (*n* = 2871), Embase (*n* = 89), and the Cochrane Library (*n* = 8), yielding a total of 6731 studies.

Prior to screening, duplicate studies (*n* = 2025) were identified and removed, leaving 4706 studies for the initial screening process. Titles and abstracts were reviewed, resulting in the exclusion of 4693 studies.

Subsequently, 13 full-text studies were retrieved and assessed for eligibility. Of these, four were excluded due to irrelevance to the research topic (*n* = 1), incomplete data (*n* = 1), and incorrect study design (*n* = 2).

**Figure 1:**
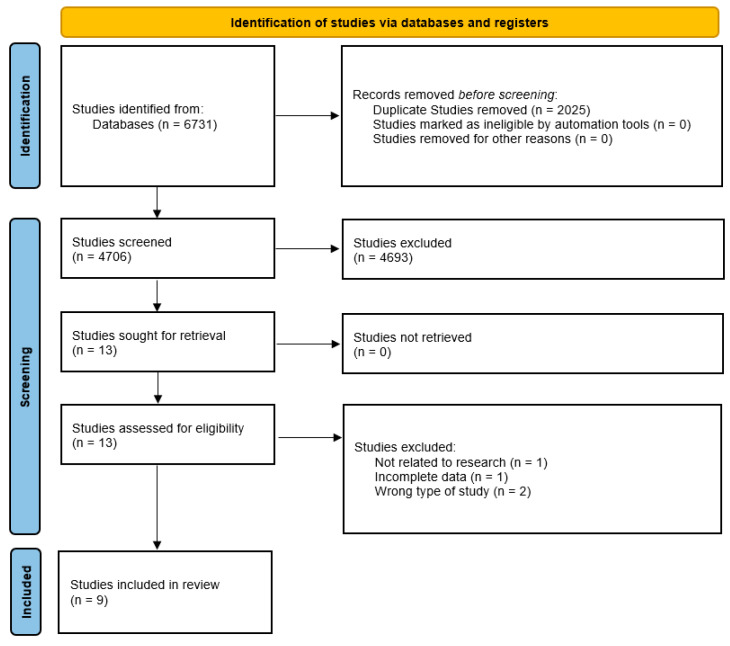
PRISMA Flowchart.

Ultimately, nine studies met the inclusion criteria and were included in the systematic review (Figure 1).

**Figure 2:**
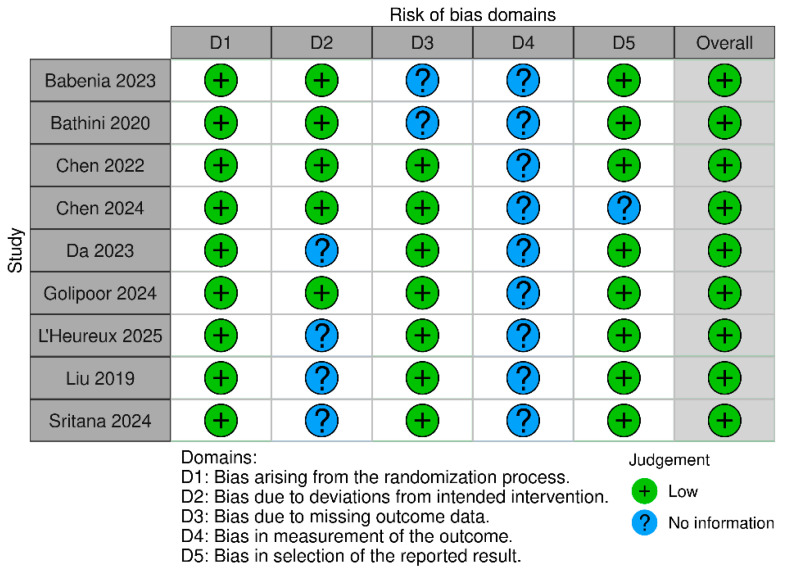
Risk of bias assessment (ROB 2) results chart [17,21,22,23,24,25,26,27,28].

The ROB 2 risk of bias assessment revealed variability in study quality. Most studies had low risk of bias in randomization and handling of missing data, although some showed moderate concerns due to unclear group allocation and participant follow-up. Potential bias in outcome measurement was identified, mainly due to a lack of blinding of assessors. Additionally, bias in the selection of reported results was noted, as some studies lacked predefined protocols. Overall, while good practices were maintained in randomization and intervention adherence, improvements in transparency, blinding, and protocol registration are needed to minimize bias (Figure 2).

The systematic review identified multiple studies investigating the relationship between oral microbiota dysbiosis and cognitive impairment, particularly Alzheimer’s disease (AD) and mild cognitive impairment (MCI). Across the included studies, various methodologies were employed to assess microbial composition, including 16S rRNA sequencing, polymerase chain reaction (PCR), and fungal culture techniques.

In 2023, Babenia [21] detected *Tannerella forsythia* and *Fusobacterium nucleatum* in 100% of AD patients, suggesting a potential link between oral dysbiosis and dementia pathogenesis. Similarly, Bathini [22] (2020) reported a stage-specific alteration in salivary microbiota, characterized by a reduction in periodontal bacteria and an increase in opportunistic species during disease progression. In 2024, Chen [24] identified *Porphyromonas*, *Prevotella*, and *Fusobacterium* as dominant genera in cognitively impaired individuals, supporting their potential as biomarkers for cognitive deterioration.

The impact of oral health interventions on cognitive function was explored by Chen [23] (2022), who observed an improvement in microbial composition and a reduction in cognitive decline among AD patients following a six-month intervention.

Mild cognitive impairment was also associated with distinct microbial profiles. Da [25] (2023) found significant alterations in the salivary microbiota of MCI patients, with increased *Veillonella* and decreased *Gemella haemolysans* and *Streptococcus gordonii*, indicating their potential as early indicators of cognitive decline. In 2025, L’Heureux [14] reported an enrichment of *Porphyromonas* and *Prevotella intermedia* in MCI individuals, particularly those carrying the APOE4 allele, while *Neisseria* and *Haemophilus* were associated with better cognitive function.

A decline in microbial diversity was also observed in AD patients. Liu [26] (2019) documented reduced salivary microbial diversity, with an increase in *Moraxella* and a decrease in *Rothia*. Fungal dysbiosis was likewise observed; Golipoor [27] (2024) identified a higher prevalence of *Candida spp.* in AD patients, alongside lower fungal diversity, suggesting a potential role for fungal colonization in disease progression. Sritana [17] (2024) further reported an enrichment of *Fusobacteriota* and *Peptostreptococcaceae* in AD patients, reinforcing the hypothesis of an oral microbial contribution to neurodegeneration.

Overall, these findings indicate that specific microbial shifts in the oral cavity may be associated with cognitive impairment, highlighting their potential utility as diagnostic biomarkers or therapeutic targets (Table 2). A recurrent observation across studies was the increased presence of periodontopathogenic bacteria such as *Tannerella forsythia*, *Fusobacterium nucleatum*, *Porphyromonas*, and *Prevotella* in individuals with AD and MCI [21,22,24]. These taxa were frequently linked to disease progression, especially among APOE4 carriers [14].

Conversely, taxa typically associated with healthy oral and cognitive status, including *Streptococcus gordonii*, *Gemella haemolysans*, *Rothia*, *Neisseria*, and *Haemophilus*, were consistently reported in lower abundance among cognitively impaired individuals [14,25,26].

Finally, the potential of oral health interventions to restore microbial balance and improve cognitive outcomes was supported by Chen [23], who demonstrated positive effects following a structured six-month intervention in AD patients. These findings collectively support the role of oral microbiota as both a marker and modifiable factor in cognitive impairment (Table 2).

### 3.1. Summary of Study Designs and Their Impact on Research Robustness

In the present systematic review, nine included studies were classified according to their research design. The majority followed an observational approach, with only one study applying an interventional model. The designs were grouped as follows.

#### 3.1.1. Cross-Sectional Observational Studies (*n* = 6)

Studies by Babenia [21] (2023), Bathini [22] (2020), Golipoor [27] (2024), L’Heureux [14] (2025), Sritana [17] (2024), and Da [25] (2023) employed a cross-sectional observational design. These investigations analyzed the oral microbiome composition at a single time point, comparing participants with AD, MCI, and healthy controls. Although this design is practical for detecting associations and differences in microbial profiles, it limits causal inference due to its inability to establish temporal relationships or track changes over time. Consequently, while these studies offer valuable descriptive data and preliminary evidence of dysbiosis in AD, they contribute moderate-quality evidence within the hierarchy of research designs.

**Table 2:** Overview of studies investigating the association between oral microbiota dysbiosis and cognitive decline.

Author/ Year	Country	N	*n* with CI *	*n* Without CI	Age Range	Type of CI Evaluated/ Stage, Grade	Neuropsychological Scales	Oral Microbiota Assessment Method	Sample Type	Bacteria Associated with Dysbiosis (Increase)	Bacteria Associated with Dysbiosis (Reduction)	Outcomes
Babenia [21] 2023	Ukraine	27	27		No age range or average age is specified.	Alzheimer’s/ it does not specify differentiated clinical stages or RDA or MMSE values.	Not reported	Polymerase chain reaction (PCR)	Gingival fluid from periodontal pockets	Tannerella forsythia, Fusobacterium nucleatum, Aggregatibacter actinomycetemcomitans	Porphyromonas gingivalis	*Tannerella forsythia* and *Fusobacterium nucleatum* were detected in 100% of Alzheimer’s patients. The microbial composition suggests a possible link between oral dysbiosis and dementia pathogenesis.
Bathini [22] 2020	Switzerland	80	38	42	CNh: 67.0 ± 9.2 years CNr: 68.1 ± 10.0 years MCI: 73.2 ± 8.1 years AD: 71.1 ± 6.6 years	Alzheimer’s/ inclusion of patients in moderate-severe phase of AD.	Mini-Mental State Exam (MMSE), Clinical Dementia Rating (CDR), University of Pennsylvania Smell Identification Test (UPSIT) CNh (cognitively normal healthy): MMSE ≈ 28.4; CDR ≈ 0.0 CNr (cognitively normal at risk): MMSE ≈ 28.4; CDR ≈ 0.1 MCI: MMSE ≈ 22.5; CDR ≈ 0.8 AD: MMSE ≈ 14.2; CDR ≈ 1.4	16S rRNA Sequencing (Illumina MiSeq)	Saliva	Leptotrichia wadei (MCI), Cardiobacterium valvarum (AD)	Filifactor villosus, Filifactor alocis, Prevotella tannerae (MCI y AD), Porphyromonas gingivalis (MCI)	The salivary microbiota presents specific changes depending on the stage of dementia, with a reduction in periodontal bacteria and an increase in opportunistic species in the progression of the disease.
Chen [23] 2022	China	66	66		82.85 ± 6.00 years	All patients were diagnosed with mild Alzheimer’s disease	Mini-Mental State Examination (MMSE), Neuropsychiatric Inventory (NPI), Nursing Home Adjustment Scale (NHAS), Alzheimer’s Disease Cooperative Study-ADL (ADCS-ADL), Kayser-Jones Brief Oral Health Status Examination (BOHSE)	16S rRNA Sequencing (Illumina MiSeq)	Subgingival biofilm	Alphaproteobacteria, Betaproteobacteria, Flavobacteria	Actinobacteria, Spirochaete, Synergistetes	The oral health intervention improved the oral microbiota and reduced cognitive impairment in patients with mild Alzheimer’s after 6 months of follow-up.
Chen [24] 2024	China	165	125	40	Normal controls: 67.45 ± 8.36 years SCD: 66.90 ± 7.98 years MCI: 66.33 ± 8.83 years Dementia: 68.44 ± 6.71 years	It does not report specific data on stage or severity, it only compares individuals with Alzheimer’s.	Mini-Mental State Examination (MMSE), Montreal Cognitive Assessment (MoCA)	16S rRNA Sequencing (Illumina MiSeq)	Subgingival biofilm	Porphyromonas, Prevotella, Fusobacterium, Leptotrichia, Campylobacter, Selenomonas	Capnocytophaga, Saccharibacteria_genera_incertae_sedis, Lautropia, Granulicatella	Subgingival microbial composition is associated with different levels of cognitive function, suggesting a possible use as a biomarker of cognitive decline.
Da [25] 2023	China	94	47	47	Not explicitly reported.	No classification is specified in degrees of severity; it is only reported as Alzheimer’s or MCI vs. controls.	Mini-Mental State Examination (MMSE), Auditory Verbal Learning Test, Trail-Making Test, RMB (Renminbi) Test	16S rDNA Sequencing (Illumina MiSeq)	Unstimulated saliva	Veillonella unclassified_Veillonella, Fusobacterium sp._HMT_203	Gemella haemolysans, Streptococcus gordonii	Older adults with mild cognitive impairment have an altered oral microbial composition compared to cognitively normal individuals. *Gemella haemolysans* and *Streptococcus gordonii* may be potential indicators of MCI.
Golipoor [27] 2024	Iran	152	76	76	AD: 87.96 ± 7.91 years Non-AD: 85.18 ± 5.79 years	Alzheimer’s/ preclinical. Very mild cognitive decline/ mild to moderate cognitive decline and severe dementia.	Global Deterioration Scale (GDS)	Fungal culture on agar Sabouraud Chloramphenicol and PCR-Sequencing	Oral mucus swab	Candida albicans (AD: 80%, No AD: 40%), Candida glabrata (AD: 9%)	Increased fungal diversity in individuals without AD	Patients with AD have a higher prevalence of *Candida* spp. and a lower fungal diversity in the oral microbiota. Fungal microbiota analysis could be an early marker of the disease.
L’Heureux [14] 2025	United Kingdom	115	55	60	Inclusion criteria: ≥50 years old. No mean age is reported.	Patients were classified as mild cognitive impairment (MCI). Patients with clinical Alzheimer’s were not included.	Mini-Mental State Examination (MMSE), Switching Stroop, Trail Making, Digit Span	16S rRNA sequencing	Mouth rinse	Porphyromonas (MCI), Prevotella intermedia (APOE4+)	Neisseria, Haemophilus (associated with better cognition)	The oral microbiome of people with MCI exhibits a higher abundance of *Porphyromonas* and a lower level of Neisseria and *Haemophilus*, suggesting a link between oral dysbiosis and cognitive decline.
Liu [26] 2019	China	78	39	39	The average age is not specified.	Alzheimer’s	Mini-Mental State Examination (MMSE), Neuropsychiatric Inventory (NPI), Clinical Dementia Rating (CDR), Activity of Daily Living Scale (ADL)	16S rRNA sequencing	Unstimulated saliva	Moraxella, Leptotrichia, Sphaerochaeta	Rothia	Patients with AD have lower salivary microbial diversity and alterations in bacterial composition, with an increase in *Moraxella* and a reduction in *Rothia*.
Sritana [17] 2024	Thailand	100	56	44	AD: 66.90 ± 7.06 years MCI: 68.50 ± 6.35 years Controls: 64.73 ± 4.78 years	Alzheimer’s/ average values are reported for AD patients.	Clinical Dementia Rating (CDR), Montreal Cognitive Assessment (MoCA), Mini-Mental State Examination (MMSE)	16S rRNA Sequencing (PacBio SMRT)	Saliva	Fusobacteriota, Peptostreptococcaceae	Veillonella	Patients with AD show greater diversity of oral microbiota and elevated levels of *Fusobacteriota* and *Peptostreptococcaceae*, suggesting a possible role in the pathogenesis of the disease.

Legend: * CI = Cognitive Impairment. The reviewed studies consistently highlighted a significant association between oral microbiota dysbiosis and cognitive impairment, particularly Alzheimer’s disease (AD) and mild cognitive impairment (MCI). Across the nine included studies, specific bacterial and fungal shifts were identified in individuals with cognitive decline, supporting the hypothesis that oral microbial alterations could contribute to neurodegenerative processes.

#### 3.1.2. Case–Control Studies (*n* = 2)

Liu [26] (2019) and Chen [24] (2024) conducted case–control studies that compared the oral microbiome of individuals with AD or MCI against cognitively healthy controls. This design provides stronger evidence than purely descriptive cross-sectional studies as it allows for targeted investigation of potential risk factors; however, it remains vulnerable to selection and recall biases. These studies strengthen the evidence base by reinforcing observed microbial patterns associated with cognitive decline.

#### 3.1.3. Randomized Controlled Trial (*n* = 1)

Only Chen [23] (2022) applied an interventional design, conducting a randomized controlled trial (RCT) to assess the effect of oral health interventions on cognitive status and oral microbiota composition in patients with mild AD. As RCTs represent the gold standard for clinical research due to their ability to minimize bias and confounding factors, this study provides the highest level of evidence among the included articles. However, the isolated presence of a single RCT limits the generalizability and overall strength of causal conclusions across the full body of evidence.

## 4. Discussion

The reviewed studies consistently highlighted a significant association between oral microbiota dysbiosis and cognitive impairment, particularly AD and MCI. Across the nine included studies, specific bacterial and fungal shifts were identified in individuals with cognitive decline, supporting the hypothesis that oral microbial alterations could contribute to neurodegenerative processes.

A recurrent finding was the increased presence of periodontopathogenic bacteria in individuals with AD and MCI. Babenia et al. [21] detected *Tannerella forsythia* and *Fusobacterium nucleatum* in all AD patients, reinforcing the role of these bacteria in oral dysbiosis linked to neurodegeneration. Similarly, Bathini et al. [22] and Chen et al. [24] reported an increase in *Porphyromonas*, *Prevotella*, *Fusobacterium*, and *Leptotrichia* in cognitively impaired individuals, highlighting their potential involvement in disease progression. In line with these findings, Morales et al. [28] reported a significant association between oral microbiota dysbiosis and Alzheimer’s disease, highlighting an increase in pro-inflammatory bacteria such as *Prevotella* and a decrease in anti-inflammatory bacteria such as Proteobacteria in patients with Alzheimer’s disease. This suggests a possible link with the onset and severity of dementia.

Additionally, L’Heureux et al. [14] found higher levels of *Porphyromonas* and *Prevotella intermedia* in MCI patients, particularly among those carrying the APOE4 allele, suggesting a possible genetic–microbial interaction. This is partially consistent with the study by Jungbauer et al. [29], who suggest that the impact of oral microorganisms, particularly *Porphyromonas*, on Alzheimer’s disease remains unclear; however, the evidence indicates a possible association, as microbial dysbiosis and inflammation may contribute to the pathogenesis and cognitive decline of the disease. Moreover, the previously mentioned study by Morales et al. [28] also supports these findings in the case of *Prevotella*.

Conversely, studies consistently reported a reduction in certain bacterial taxa associated with healthy oral and cognitive status. Da et al. [25] and Liu et al. [26] observed a decline in *Streptococcus gordonii*, *Gemella haemolysans*, and *Rothia* among cognitively impaired patients. Additionally, L’Heureux et al. [14] found lower levels of *Neisseria* and *Haemophilus*, which were associated with better cognitive function, reinforcing the hypothesis that a balanced oral microbiota might play a protective role in maintaining cognitive health.

Beyond bacterial shifts, fungal dysbiosis was also observed in AD patients. Golipoor et al. [27] identified a significantly higher prevalence of *Candida albicans* and *Candida glabrata* in AD patients, coupled with a reduced overall fungal diversity, suggesting that fungal colonization may contribute to neurodegeneration.

Among the included studies, Liu [26] (2019) uniquely reported no association between oral microbiota composition and clinical severity of AD. Additionally, no standardized cognitive scales such as Clinical Dementia Rating or Mini-Mental State Examination were documented in that study. This absence of clinical severity data introduces a methodological limitation when integrating findings across studies. Most other included investigations established either direct (Bathini [22] 2020, Sritana [17] 2024) or indirect (Golipoor [27] 2024) associations between microbial shifts and disease staging.

Despite these findings, studies such as those by Wan and Fan Wan and Fan [30] and Dominy et al. [15] support the notion that a relationship may exist between certain microorganisms present in the oral microbiota and the onset or progression of dementia, particularly among older adults. Conversely, a systematic review conducted by Pruntel et al. [31] reported that there is no conclusive evidence linking a specific oral microbiota composition to Alzheimer’s disease, with many studies reporting contradictory results. Therefore, it remains uncertain whether specific oral microorganisms are associated with the development or worsening of dementia.

Furthermore, another study by McGuinness et al. [32] found no significant associations between periodontitis and dementia or mild cognitive impairment. In addition, no differences were observed in serum IgG titers against periodontal pathogens among patients diagnosed with dementia or mild cognitive impairment compared to those with normal cognition.

Moreover, the influence of oral health interventions on microbiota and cognitive outcomes was demonstrated by Chen et al. [23], who reported improvements in microbial balance and cognitive function following a six-month oral health intervention in AD patients. These findings support the potential for targeted oral health strategies to mitigate cognitive decline.

A recent Korean study reported significant differences in both the diversity and composition of the oral microbiome between older adults with dementia and cognitively healthy individuals, suggesting its potential as a diagnostic biomarker [33]. Similarly, another investigation identified distinct salivary microbiome and proteome signatures capable of differentiating between individuals with mild cognitive impairment, dementia, and healthy controls [34].

A comprehensive review highlighted the role of oral microbiota in Alzheimer’s pathology by integrating modifiable risk factors and describing pathways involving systemic inflammation, blood–brain barrier disruption, and oxidative stress [35]. Furthermore, a systematic analysis specifically examined the relationship between periodontitis, the oral microbiome, and Alzheimer’s disease, supporting the contribution of chronic oral infections to neurodegenerative progression [36].

Pharmacological treatments commonly used in dementia, such as donepezil and memantine, have been shown to significantly alter the composition of the salivary microbiome, particularly in patients with underlying neurodegenerative conditions [37].

Evidence from patients with Parkinson’s disease and cognitive impairment revealed virulence-associated microbial signatures along the oral–gut–brain axis that may influence disease pathophysiology [38]. Additionally, a study conducted among Hispanic individuals identified distinct oral and gut microbiome profiles between cognitively impaired and unimpaired participants [39].

The reviews by Liu et al. [40] and de Tao et al. [41] reported inconsistent findings regarding bacterial communities in the oral microbiota and their association with Alzheimer’s disease. They noted that the relationship between the oral microbiota and neuropsychiatric disorders, including dementia, is complex and not yet fully understood. While certain microorganisms may influence mental health, specific connections with dementia require further research and have not been definitively established.

Taken together, these results suggest that oral microbiota dysbiosis is not only a consequence of cognitive decline but may also play a contributory role in its pathogenesis. The consistent presence of periodontal pathogens in AD and MCI patients, coupled with a decline in health-associated microbial species, supports the hypothesis that microbial imbalance may contribute to neuroinflammatory and neurodegenerative processes. Additionally, the observed improvements following oral health interventions highlight the potential of microbiome-targeted strategies in mitigating cognitive decline. These findings underscore the need for further longitudinal and mechanistic studies to establish causality and explore potential therapeutic applications. A recent study employed Mendelian randomization analysis to investigate the bidirectional causal relationships between eight oral health conditions and four psychiatric disorders. The results indicated that painful gums and oral ulcers are associated with an increased risk of major depression and bipolar disorder, suggesting a genetic link between oral health and mental health [42]. Additionally, a systematic review and meta-analysis identified a positive association between depression and oral diseases such as dental caries, tooth loss, and edentulism in adults and older individuals. Although most of the included studies were cross-sectional, the authors emphasized the need for longitudinal research to establish causal and temporal relationships [43].

### 4.1. Proposed Scheme on How Oral Microbial Dysbiosis May Influence the Development of Dementia

The findings of this systematic review highlight a consistent pattern of oral microbial dysbiosis associated with cognitive impairment and dementia. The available evidence suggests a multifactorial mechanistic pathway linking oral microbiota alterations to neurodegeneration.

Primarily, a shift in the oral microbial community favors the overgrowth of periodontopathogenic and opportunistic bacteria such as *Porphyromonas gingivalis*, *Tannerella forsythia*, *Fusobacterium nucleatum*, and *Prevotella* species, as shown in the included studies by Babenia et al. [21], Bathini et al. [22], Chen et al. [23], and L’Heureux et al. [14]. These pathogens may enter systemic circulation via inflamed periodontal tissues and subsequently cross the blood–brain barrier (BBB), especially in genetically susceptible individuals, such as APOE4 carriers [14].

*P. gingivalis* and its virulence factors, including gingipains and outer membrane vesicles, have been implicated in neuroinflammation and the promotion of β-amyloid plaque formation in the brain, as supported by prior mechanistic studies and observed in the reviewed articles [21]. The increase in Fusobacteriota and Peptostreptococcaceae in AD patients, as reported by Sritana et al. [17], further supports the hypothesis of oral dysbiosis contributing to neuroinflammation and neuronal injury through bacterial endotoxins and inflammatory mediators [17].

In parallel, a reduced abundance of commensal taxa such as *Neisseria*, *Haemophilus*, *Rothia*, *Streptococcus gordonii*, and *Gemella haemolysans*, as demonstrated by Da et al. [25] and Liu [26], may impair oral mucosal immunity and nitric oxide metabolism, weakening the host defense and facilitating pathogen invasion [24,25,27]. Fungal dysbiosis, characterized by the overgrowth of *Candida albicans* and *Candida glabrata*, may further amplify inflammatory responses and exacerbate BBB dysfunction [27].

Collectively, these microbial changes may promote systemic inflammation and neuroimmune modulation, leading to impaired amyloid clearance, tau hyperphosphorylation, synaptic dysfunction, and progressive cognitive decline. Notably, Chen et al. [23] showed that targeted oral health interventions can partially restore microbial balance and attenuate cognitive deterioration, suggesting a potential therapeutic window.

Oral microbial dysbiosis may influence dementia development through (1) microbial translocation and neuroinvasion, (2) systemic and neuroinflammation, and (3) the disruption of protective microbial communities and host–microbiome interactions. This complex interplay, modulated by genetic and environmental factors, provides a compelling biological rationale for the association observed in this systematic review.

### 4.2. Limitations of the Study

The sample size was relatively small, which may have limited the statistical power of the findings and the generalizability of the results. Future studies with larger cohorts are needed to confirm these observations.

While 16S rRNA sequencing was used to analyze microbial composition, this technique has limitations in species-level resolution. Whole-genome sequencing or metagenomic approaches could provide more detailed insights into microbial functions and interactions.

Additionally, variations in oral hygiene practices, dietary habits, and previous dental treatments among participants may have influenced microbiome composition. Although efforts were made to control these factors, residual confounding cannot be ruled out.

The predominance of observational designs (eight out of nine studies) highlights that current research on the association between oral microbiota dysbiosis and dementia risk is primarily exploratory and descriptive. While these studies consistently document differences in microbial profiles, the lack of longitudinal cohort studies and the scarcity of controlled trials reduce the certainty regarding causality. Therefore, although the evidence supports a potential relationship between oral dysbiosis and dementia, further longitudinal and interventional research is needed to confirm causation and assess clinical applicability.

Despite these limitations, the study provided valuable insights into the potential relationship between oral microbiota and cognitive impairment, highlighting the need for further research in this area.

## 5. Conclusions

Key findings showed that individuals with mild cognitive impairment and Alzheimer’s disease exhibited significant differences in the diversity and abundance of oral bacteria compared to cognitively healthy individuals. Specifically, a decrease in bacterial richness was observed as cognitive impairment advanced, with a significant increase in pathogenic bacteria such as *Porphyromonas*, *Fusobacterium*, and *Peptostreptococcaceae*, and a decrease in beneficial bacteria, including *Capnocytophaga*, *Veillonella*, and *Gemella haemolysans*.

Early identification and intervention targeting oral dysbiosis may represent a promising strategy for dementia prevention and treatment, emphasizing the importance of oral health as an integral component in the clinical management and approach of older patients at risk or in the early stages of cognitive impairment.

## Figures and Tables

**Table 1:** Search strategies for each database used in the review.

Database	Formulation	Filters
Pubmed	((“Oral microbiome” [Title/Abstract] OR “oral microbiota” [Title/Abstract] OR “oral bacteria” [Title/Abstract] OR “oral dysbiosis” [Title/Abstract]) AND (“Dementia” [Title/Abstract] OR “cognitive decline” [Title/Abstract] OR “Alzheimer’s disease” [Title/Abstract] OR “neurodegeneration” [Title/Abstract] OR “mild cognitive impairment” [Title/Abstract])) OR ((“Microbiota” [MeSH Terms] OR “Oral microbiome” [MeSH Terms] OR “Bacteria” [MeSH Terms]) AND (“Dementia” [MeSH Terms] OR “Alzheimer Disease” [MeSH Terms] OR “Neurodegenerative Diseases” [MeSH Terms]))	Clinical Study, Clinical Trial, Randomized Controlled Trial.
Scopus	(TITLE-ABS-KEY (“oral microbiome” OR “oral microbiota” OR “oral bacteria” OR “oral dysbiosis” OR “oral microbial community” OR “oral flora” OR “oral microbial diversity” OR “oral microbial ecosystem” OR “oral microbial composition” OR “oral microbiological profile” OR “oral pathogens” OR “oral microorganisms” OR “microbiota” OR “bacteria”) AND TITLE-ABS-KEY (“dementia” OR “cognitive decline” OR “Alzheimer’s disease” OR “neurodegeneration” OR “mild cognitive impairment” OR “neurocognitive disorders” OR “cognitive dysfunction” OR “neurodegenerative diseases” OR “cognitive disorders” OR “brain aging”))	AND (LIMIT-TO (DOCTYPE, “ar”))
WoS	(TS = (“oral microbiome” OR “oral microbiota” OR “oral bacteria” OR “oral dysbiosis” OR “oral microbial community” OR “oral flora” OR “oral microbial diversity” OR “oral microbial ecosystem” OR “oral microbial composition” OR “oral microbiological profile” OR “oral pathogens” OR “oral microorganisms” OR “microbiota” OR “bacteria”) AND TS = (“dementia” OR “cognitive decline” OR “Alzheimer’s disease” OR “neurodegeneration” OR “mild cognitive impairment” OR “neurocognitive disorders” OR “cognitive dysfunction” OR “neurodegenerative diseases” OR “cognitive disorders” OR “brain aging” OR “cognitive impairment”))	Refined By:Document Types: Article
Embase	(‘oral microbiome’: ti,ab,kw OR ‘oral microbiota’: ti,ab,kw OR ‘oral bacteria’: ti,ab,kw OR ‘oral dysbiosis’: ti,ab,kw) AND (dementia: ti,ab,kw OR ‘cognitive decline’: ti,ab,kw OR ‘alzheimers disease’: ti,ab,kw OR neurodegeneration: ti,ab,kw OR ‘mild cognitive impairment’: ti,ab,kw)	
Cochrane Library	(“Oral microbiome” OR “oral microbiota” OR “oral bacteria” OR “oral dysbiosis”): ti,ab,kw AND (“Dementia” OR “cognitive decline” OR “Alzheimer’s disease” OR “neurodegeneration” OR “mild cognitive impairment”): ti,ab,kw (Word variations have been searched)	

## Data Availability

All data extracted from the included articles, data processing, and screening are available in the data repository through this dataset: https://doi.org/10.17632/h48gz4hcd8.1 (accessed on 18 April 2025).
